# “Learning a Way of Thinking”—World Café on Clinical Reasoning in Nursing and Midwifery Education and Practice across Five European Union Countries

**DOI:** 10.3390/healthcare11222969

**Published:** 2023-11-16

**Authors:** Ljubiša Pađen, Manca Pajnič, Renata Vettorazzi, Ana Pérez-Perdomo, Małgorzata Stefaniak, Nele Claes, Hugo Franco, An Vandervoort, Mirjam Ravljen

**Affiliations:** 1 Faculty of Health Sciences, University of Ljubljana, 1000 Ljubljana, Slovenia; ljubisa.paden@zf.uni-lj.si (L.P.); manca.pajnic@zf.uni-lj.si (M.P.); renata.vettorazzi@zf.uni-lj.si (R.V.); 2 Hospital Clinic of Barcelona, 08036 Barcelona, Spain; anperez@clinic.cat; 3 Faculty of Health Sciences, Medical University of Warsaw, 02-091 Warsaw, Poland; malgorzata.stefaniak@wum.edu.pl; 4 HBO Verpleegkunde Genk, 3600 Genk, Belgium; nele.claes@verpleegopleiding-genk.be; 5 School of Health Setúbal, Setúbal Polytechnic University, 2910-761 Setúbal, Portugal; hugo.franco@ess.ips.pt; 6 UCLL Health Innovation, 3600 Genk, Belgium; an.vandervoort@ucll.be

**Keywords:** clinical reasoning, decision making, education, healthcare, participatory method, co-production

## Abstract

Clinical reasoning is a key attribute of nursing and midwifery professionals. As a part of the Erasmus plus project, we designed a study with the aim of exploring the understanding of clinical reasoning as a concept, experiences of teaching clinical reasoning and practices related to using clinical reasoning in nursing and midwifery. A qualitative study was carried out using the World Café method, involving 44 participants from five European countries. The participants represented diverse professional backgrounds, including nurses, midwives and lecturers. Our analytical approach was based on a thematic analysis. We categorized the data into three main categories, namely, “Spiral of thinking”, “The learning and teaching of a way of thinking” and “Clinical reasoning in real life”, all under an overarching theme, “Learning a way of thinking”. This study highlighted areas of learning and teaching which can be improved in current nursing and midwifery education. Furthermore, it identified barriers, facilitators and practices from five European countries which can be used in the further development of nursing and midwifery curricula and courses with the aim of enhancing clinical reasoning competence and ultimately improving patient care.

## 1. Introduction

Global challenges such as increased service requirements due to the complex health needs of people, inadequate or limited human resources, poor health systems financing, fast-paced and time-limited care and climate challenges [1,2,3,4] are guiding stakeholders to develop new strategies in order to improve healthcare access, the knowledge base and competence of health professionals and patient outcomes [5,6,7,8,9]. At the bedside, nurses and midwives who are caring for people with complex and diverse conditions must rapidly respond to challenges by making decisions to adapt their way of clinical working to achieve optimal care outcomes and reduce the risk of errors [10]. One of the approaches to improve decision making in clinical care is clinical reasoning because clinical reasoning is a process of thinking and decision making involved in clinical practice and the caring healthcare context [11,12,13]. Clinical reasoning is an ongoing process in which nurses and midwives quickly and accurately assess care situations by collecting cues, making precise observations, processing information and gaining an understanding of a person’s problem. They then plan and implement appropriate interventions, take necessary actions with specific goals in mind, evaluate outcomes and engage in reflection and learning from this process [14].

Although clinical reasoning in medical education is a well-developed and valued approach [15], it is not well integrated into nursing and midwifery curricula. A scoping review carried out by de Menezes and colleagues has shown that the majority of evidence related to clinical reasoning education in nursing and midwifery comes from the United States and other high-income countries such as the United Kingdom, Canada, Norway, Spain, Turkey, Taiwan and Korea. The authors have identified that there is a challenge related to teaching strategies and methods of clinical reasoning in nursing education [16], which is consistent with findings from a recent review carried out by Brown Tyo and McCurry [17] in which a need to further develop not only educational strategies but also an assessment method which is specific, valid and reliable is emphasized.

There is also a general lack of literature from the European Union (EU) context. In the EU, nursing and midwifery education is regulated by EU directives [18,19] which aim to harmonize education to allow for the mutual recognition of qualifications and freedom of movement for workers within the Union. To the best of our knowledge, the only qualitative study aimed at exploring barriers to teaching clinical reasoning in health professional education was carried out by Sudacka et al. [20]. The findings from this study showed that the barriers were mostly related to understanding clinical reasoning as a concept, teaching and assessment practices for clinical reasoning, a lack of material resources and cultural barriers. Interestingly, they also identified not only interprofessional but also intraprofessional differences and barriers, for example, between clinical teachers and university lecturers, which is an important finding, especially in the context of nursing and midwifery for which half of the education process is carried out in a clinical environment [18,19], which shows that clinical skills are to be gained from work experience, while hands-on training is seen as a crucial component of undergraduate nursing and midwifery courses.

Even though most of the research investigates how nurses clinically reason, unfortunately, scarce research is dedicated to questioning how nursing students develop clinical reasoning skills. A report developed by Holder [21] addressed that by researching linkages and determining how clinical reasoning develops in the student population and what factors affect this development, educators could then determine the most effective method for developing reasoning in individual students. The report “*Transforming nursing education in response to the Future of Nursing 2020–2030*” identifies as one of the pillars of this transformation the challenge in curricular designs focusing on clinical reasoning and simulation-based learning environments [22].

To address some of the existing challenges, a consortium of partners from five EU countries—Belgium, Spain, Poland, Portugal and Slovenia—developed the Erasmus plus project Clinical Reasoning in Nursing and Midwifery Education and Practice (2022–2024). The ultimate goal of this project is to develop a framework, basic and advanced education and training and a charter in order to support nursing and midwifery professionals in enhancing their clinical reasoning abilities. To support the project objectives, we carried out a study with the aim of exploring the understanding of clinical reasoning as a concept, experiences of teaching clinical reasoning and practices related to using clinical reasoning in nursing and midwifery. The specific research questions we intended to answer with this project were as follows:

What is the understanding of clinical reasoning as a concept?

What are the experiences of teaching clinical reasoning?

What are the practices related to using clinical reasoning in nursing and midwifery?

## 2. Materials and Methods

### 2.1. Study Design

We applied a qualitative exploratory design, using a World Café as a method of data collection. The World Café method enables a process of structured learning and knowledge exchange between different stakeholders [23], and it has been successfully used for exploratory qualitative research in health and social care contexts [24,25,26]. The World Café method, in contrast to individual interviews as a data collection technique, allows for flexibility in data collection in large groups, allows participants to explore multiple perspectives and encourages them to share ideas and build on each other’s thoughts, providing a comprehensive perspective on a studied phenomenon [23].

### 2.2. Participant Recruitment

We used a convenience sampling approach as the aim of this study was exploratory in its nature and the study focused on specific population, e.g., nursing and midwifery professionals. Furthermore, we had access to recruit participants attending a clinical reasoning learning, training and teaching (LTT) course which was organized by our project partners.

Each partner project coordinator personally invited up to 10 participants with diverse professional backgrounds and a mixed range of experience (i.e., junior or senior nurses, junior or senior midwifes and nursing and midwifery lecturers at various academic levels including university professors, unit managers, and nursing or midwifery directors). The participants, according to the inclusion criteria, had to be employed in nursing/midwifery education, research, or practice in one of the five participating countries.

### 2.3. Data Collection

The World Café was held on 6 March 2023 at University College Leuven-Limburg, Genk, Belgium (an integral part of a 5-day LTT course), and was carried out in English. We divided participants into four café table groups, each covering three themes, namely, defining clinical reasoning, teaching clinical reasoning and using clinical reasoning in clinical practice. Each thematic session started with presenting the participants with the objectives of the session and guidance related to communication and mutual respect, ensuring a safe environment for participants to share their opinions and practices. For people who felt that they could express their opinion more thoroughly in their native language, translation by a member of the project team was offered. The facilitator of each thematic session opened the discussion with the main questions and used prompts to either clarify or further develop the discussion (the question guide and the facilitators’ characteristics are available in Appendix A). At the end of each session, the participants from each table provided feedback to the World Café. The participants then rotated to another café table for the next thematic session (altogether, there were three sessions with a total duration of approximately 90 min). At the end of the World Café, conclusions were discussed. Data were collected using different techniques, namely, handwritten notes made by participants and facilitators during each thematic session, collated participant responses to key theme questions on sticky notes and posters and facilitators’ notes on the group reflection. All materials at the World Café were produced in an anonymous way (it was not possible to assign material to individuals except for the facilitators’ notes). Collected data were encoded, for example, Facilitator note 1, Participant note 1 or Group note 1.

### 2.4. Data Analysis and Rigor

Data were analyzed using a thematic analysis, as described by Braun and Clarke [27], following a six-step process: two pairs of researchers (L.P. and M.R. and M.P. and R.V.) who were skilled in qualitative methods and had a professional background in nursing, education, or organizational sciences and management familiarized themselves with the data by reading and re-reading the raw material and transcribed data, making notes of their initial impressions. Then, the researchers collated data from all sources into one file and generated initial codes, following the principle of open coding. Both pairs of researchers independently sought patterns, organizing the data into preliminary subcategories and categories aligning their relevance with the study’s objectives. During the step of defining and naming themes, we considered how the data supported each theme and whether the themes made sense. The final step, which included defining themes and producing the final report, was discussed through an online consultation with the rest of the study team (A.P.-P., M.S., N.C., H.F. and A.V.). Data were analyzed using Microsoft Excel.

The rigor of the study was ensured by applying Lincoln and Guba’s [28] trustworthiness criteria for qualitative research. We followed the COREQ EQUATOR guidelines (Supplementary Materials) [29] in reporting this study.

### 2.5. Ethics

This study followed the principles of the Declaration of Helsinki [30]. The research proposal was reviewed and approved by the Division of Nursing, University of Ljubljana (code: 2-3/22; approval date: 15 November 2022). We followed the basic principles of ethics in health science research, namely, autonomy, beneficence, non-maleficence and justice [31]. All participants received a study information sheet and provided both verbal and written consent prior to participating in this study. Furthermore, participants had the right to withdraw at any time without any consequences. We believe that this study was beneficial for participants and nursing/midwifery as a professional community as it allowed participants to exchange ideas, practices and experiences and generated new knowledge. Furthermore, participants could learn from each other in a safe and respectful environment. To ensure non-maleficence, we also protected the participants’ identities through the assurance of confidentiality and anonymity (data cannot relate to a participant’s identity). Moreover, this study explored participants’ experiences of their educational strategies and methods of teaching and learning clinical reasoning; therefore, we believe that the probability and magnitude of expected discomfort for the participants did not exceed the situations encountered in their everyday professional lives. To ensure justice, we initiated the World Café session by emphasizing mutual respect and the inclusion of all participants in sessions. Furthermore, based on our study’s aim, we included participants with various professional backgrounds related to nursing and midwifery to “give voice” to all relevant stakeholders.

Participants received an allowance for travelling to the learning, training and teaching course but did not receive any financial remuneration for participating in the study.

## 3. Findings

A total of 44 professionals (8 from Spain, 18 from Belgium, 6 from Portugal, 8 from Poland and 4 from Slovenia) participated in this study, 4 of whom were males and 40 were females. The majority of the participants (*n* = 31) had nursing backgrounds—the participants were working as nurses, nursing lecturers, or researchers (including dual roles—practice and education or research) in different fields of nursing (general and/or specialist medicine, general and/or specialist surgery, emergency and intensive care health promotion, mental health and elder care). Eleven participants were midwives working as educators, researchers and clinical midwives (including dual roles). One participant was a psychologist and one was a medical doctor.

The data were categorized into three main categories, namely, “Spiral of thinking”, “the learning and teaching of a way of thinking” and “Clinical reasoning in real life”, all under an overarching theme, “Learning a way of thinking” (Table 1).

### 3.1. Category 1: Spiral of Thinking

#### 3.1.1. Subcategory: Complexity

Clinical reasoning has been described as a complex systematic and dynamic process and understood as a way of thinking. It is encompassed by a phased approach of gathering information, identifying problems and analyzing and organizing information into meaningful units of information which is followed by reasoning about information, setting priorities, and decision making in relation to clinical care.

Clinical reasoning was described by participants *as creating a “picture” and solving a “puzzle” (Participant note 37)* about clinical problems by using *an “upward spiral and reverse-spiral analytical approach” (Participant note 28)*. The upward spiral builds a clinical picture from collected data about patients and is therefore inductive, while the reverse-spiral approach is understood as deductive reasoning and challenges linearity and dualistic thinking in reasoning.

#### 3.1.2. Subcategory: Meta-Skills and Competency

Participants reflected that clinical reasoning skills are developed by intertwining a wide range of “meta” competencies, such as professional values, emotional and social intelligence, cognitive abilities, communication, analytical skills and problem solving, and reflective practice.

Competence in clinical reasoning develops and progresses through time along with clinical expertise. Students and novice practitioners need to approach systematically reasoning and making decisions related to patient care. Experts can rely on their knowledge, which is informed by high-certainty evidence, past clinical experience (clinical expertise) and ethical competencies and acts as a reservoir of knowledge and a foundation for reasoning and making ethical clinical decisions.

*Novice nurses, due to a lack of experience, can miss important data about patients, while experts have a lot of experience and have developed a gut feeling. Clinical reasoning develops with experience, not only in a professional sense but also personal.*

(Facilitator 3 note 5)

### 3.2. Category 2: The Learning and Teaching of a Way of Thinking

#### 3.2.1. Subcategory: Benefits and Challenges

The participants discussed the benefits of learning and teaching clinical reasoning. Apart from the goal of students developing skills for making correct decisions, participants also highlighted how students develop and enhance transferable skills and underlying learning areas, namely, developing clinical intuition, acquiring and organizing knowledge, identifying and reflecting on their own perspectives, experiences and biases, learning to work in different clinical environments or teams with different dynamics and cultures, learning how to interact with patients and co-workers and learning to communicate in difficult circumstances. Transferable skills and underlying learning areas feed the process of students developing the capacity to reason effectively.

*Teaching clinical reasoning is not a standalone theoretical teaching unit; it is much broader and deeper. It also involves generic and soft skills that will help students reason in their practice.*

(Group 1, note 2)

Challenges related to teaching and learning clinical reasoning mostly relate to the experiences and practices of teachers from their own teaching context. The participants noted that challenges could relate to lack of teacher expertise in teaching clinical reasoning. Furthermore, some participants reported that there is a general lack of training events in clinical reasoning related to nursing and midwifery rather than to medicine.

*It is hard to find accessible training events in clinical reasoning. There are some online, but mostly in medical faculties.*

(Facilitator 1 note 10)

Another challenge in teaching clinical reasoning is the learning environment. Participants reported that a gradual (simple to complex), step-by-step approach would be best suited to teaching clinical reasoning. Teaching and learning clinical reasoning should start in a “safe” environment such as a *simulation skills labs or classroom (Group 2 note 2*); however, they also noted that this might be a challenge as *nursing and midwifery students have their clinical training in very early stage of their studies (Group 3 note 4*). Furthermore, students are taught by clinical mentors and supervisors who are clinical experts, which was perceived both as a facilitator of learning and also as a barrier as clinical experts are not trained in teaching clinical reasoning and therefore often do not address all relevant learning areas.

Resources were a prominent part of the discussion relating to challenges, specifically material resources. The participants were well aware of the availability of digital teaching materials such as virtual simulations and games, but due to a lack of resources, such materials were not accessible to them.

*Advanced simulations of procedures require material, human and financial resources which are not available to everyone.*

(Group 3 note 6)

Furthermore, there is a gap between countries and environments in terms of the possibilities of carrying out software, hardware and in situ simulations which is not only related to material resources but also the fact that the curriculum is already full of required content, and it is not possible to add new content with advanced teaching methods.

*The theoretical part of midwifery curriculum is full, and it is hard to add new content.*

(Group 4 note 2)

#### 3.2.2. Subcategory: Implementing Clinical Reasoning in Curricula

Clinical reasoning should be embedded throughout complete nursing and midwifery education as it allows healthcare professionals to *bring together the theory and practice* (*Facilitator 2 note 8*). Participants described various approaches to and factors in successfully implementing clinical reasoning into a nursing and midwifery curriculum. When considering formally teaching clinical reasoning, a framework, content and goals should be established.

*Different frameworks of clinical reasoning can be used, for example Levett-Jones, Bakker or nursing process as a way of thinking and working in nursing.*

(Group 1 notes 6–9)

The content and goals of teaching units should be related to nursing and midwifery phenomena, address theoretical knowledge and follow the principles of holistic nursing and midwifery care.

*It is important to move away from a strict biomedical approach, for example teaching dualistic vision (is present, is not present) as nursing is much more than illness and medications.*

(Group 4 notes 6)

Furthermore, clinical reasoning is often used and taught using examples of urgent situations, which fails to recognize the depth and breadth of the nursing and midwifery scope of practice.

Methods of teaching should be flexible and must support learning goals which extend beyond learning factual knowledge about a condition or illness and also support, on one hand, a structured way of thinking and the development of meta-skills such as *communication, reflection, compassion, and awareness of one’s own bias, to support one’s thinking processes. (Group 1–4)*. It should also enhance proactive—and not only reactive—knowledge, skills and behaviors. The examples of teaching methods which were provided by the participants were student-centric and involved experiential learning, participative learning and problem-solving methodologies such as *simulation, experience learning in real-life cases, clinical placements, video games/scenarios, virtual patients, videos/films, case studies and writing case reports, MM conferences, observations, self-debriefing and reflection, and dialogue with peers, supervisors and teachers (Group 1–4).* The participants suggested that apart from using different tools and scores to support the structured learning of clinical reasoning, such as *ABCDEF, TALK, ISBAR and BRADEN, WATERLOW,* and *GCS (Facilitator 1–4),* other approaches, for example, *patient biography and patient narrative, could also be useful as it creates a holistic “picture” (Facilitator 4 notes 2).* Furthermore, integrating an interprofessional teaching and learning approach is/would be of added value.

*It would be great if students could learn together with students from other disciplines.*

(Group 1 note 5)

When deciding on a teaching method, teachers should consider student characteristics, the availability of resources and the environment in which the learning is to take place.

Assessment methods should be carefully considered when implementing clinical reasoning in a curriculum. The emphasis is that the method should assess the set learning objectives and goals. Participants suggested using *“soft” and “less-formal” approaches such as reflection and debriefing or using checklists rather than written or oral exams (Group 2 note 5).*

### 3.3. Category 3: Clinical Reasoning and Decision Making in Real Life

#### 3.3.1. Subcategory: Levels and Autonomy of Decision Making

Participants reflected on how nurses and midwives reason in their clinical practice. It was noted that nurses and midwives reason and make various levels of decisions with different degrees of autonomy. Participants reported that nurses and midwives autonomously reason in the field of nursing and midwifery (as a part of the nursing process); however, they also emphasized that nurses and midwives share their concerns about patients’ problems with other members of a team (mostly physicians) and share decision making with them. *Nurses and midwives recognize the deteriorating conditions and alarm physicians, and they often act together. (Group 1 note 11).* Working together as part of a team was recognized as an essential attribute in clinical reasoning and decision making.

The way nurses reason and make decisions was noted to be associated with the area and the environment of nursing and midwifery practice. Participants discussed how clinical reasoning happens at different paces in different environments. One group noted that *working in a preventive care setting compared with intensive care allows you to consider all the information much more thoroughly, and as a nurse you don’t need to make rapid decisions (Facilitator 3, note 7).* Moreover, it was also noted that the reasoning process relies on whatever information is available, and in urgent care, nurses and midwives often do not have all the information which could be relevant for decisions regarding patient care.

#### 3.3.2. Subcategory: Clinical Reasoning as an Integral Part of Holistic Care

The participants made it clear that the use of clinical reasoning in nursing and midwifery is not only restricted to solving acute problems but is used in different contexts of health care.

*Clinical reasoning is not used only in urgent care but also in positive health care.*

(Group 4 note 9)

The participants emphasized that clinical reasoning in nursing and midwifery’s scope of practice is used in addressing patients’ physical, psychological, social and spiritual domains of health as these are interconnected and form the uniqueness of human existence. With a holistic perspective, nurses and midwives can effectively reason about all of a patient’s relevant needs and not only their physical ones. *Clinical reasoning in nursing is biopsychosocial and not only physical (Participant note 12). Reasoning is holistic (Participant note 24). It’s about the person as a whole (Participant note 47).* The participants also reflected that nurses and midwives reason in relation to and with patients, patients’ loved ones and the community within their scope of practice.

## 4. Discussion

This study’s aim was to explore the understanding of clinical reasoning as a concept, experiences of teaching clinical reasoning and practices of using it in nursing and midwifery. Three categories were developed during the analytical process, namely, *spiral of thinking, learning and teaching a way of thinking and clinical reasoning in real-life*.

The first category, *spiral of thinking*, illustrates the understanding of clinical reasoning as a concept. It emphasizes the complexity of the process and meta-skills which underpin the reasoning itself. Furthermore, “Spiral” reflects the layers of factors that are interconnected in ways and with effects which are yet to be established. There is a resemblance with the definitions and frameworks of clinical reasoning proposed by Levett Jones [14], Johnson and Webber [32] and Baker et al. [33]. However, this study further explains clinical reasoning as a complex process which is not an isolated ability but built upon healthcare professionals’ meta-cognition, prior experience, skill sets and competencies in general.

In the first rounds of discussion, some participants had difficulty when attempting to explain what clinical reasoning is. As the discussion progressed, they gained insight and recognized it as a concept (an “Ahah!” moment) and were able to discuss it further. Some participants also reflected that clinical reasoning is understood as a synonym for critical thinking or decision making, which is similar to findings by Hong et al. [34]. Although thinking, reasoning and decision making are closely interconnected processes within the real-world healthcare context, there is a theoretical conceptual difference between all three. Critical thinking serves as a form of supportive thought in the process of clinical reasoning, with decision making being the outcome of that clinical reasoning [34,35]. Furthermore, there is an ongoing discussion related to the conceptualization of clinical reasoning in nursing itself: the process of clinical reasoning in nursing students involves integrating various factors such as professional standards and system requirements [36].

The category *the learning and teaching of a way of thinking* reflects the challenges, barriers and teaching practices involved in clinical reasoning. The participants, in their discussions, signaled that learning clinical reasoning is a complex task and also drew attention to general learning outcomes and transferable skills, which are often overlooked in setting education objectives but contribute to developing clinical reasoning skills.

Additional barriers highlighted by participants were resources, namely, material, human and time resources, which is similar to the findings of Sudacka et al. [20]. Both studies pointed to a need for implementing the concept of clinical reasoning in nursing and midwifery education, defining resources in which special consideration should be given to an educator’s (university lecturer or clinical teacher) competence for teaching clinical reasoning and time, which not only reflects the time for learning and teaching clinical reasoning in the curriculum itself but also the timing of when to teach clinical reasoning in different stages of the education process.

The participants also highlighted strategies for integrating clinical reasoning into nursing curriculum. Clinical reasoning in nursing education differs in Europe. Some countries integrate it across the program, while others focus on individual subjects or use problem-based learning. Specific models may or may not be preferred. The common thread is the importance of clinical reasoning in nursing education, adapted to each country’s approach. Due to the nature of nursing and midwifery education (education and training in different clinical and non-clinical environments which is guided by EU directives), the development of clinical reasoning competence requires a comprehensive strategy in which all elements of the education process, such as learning objectives, teaching strategies and assessment methods, should be considered, planned and implemented [16,20,34]. The participants had experiences of using different evidence-based approaches for teaching clinical reasoning, such as simulation and games [37,38]. However, for some of the mentioned approaches, such as Morbidity and Mortality conferences or self- and peer-debriefing, there is a general lack of evidence of effectiveness, or the evidence is inconclusive [39,40,41,42]; therefore, careful consideration is needed when planning teaching approaches and methods.

Interestingly, the participants suggested using “soft” methods for assessing clinical reasoning, which conflicts with the findings from a recent scoping review carried out by Daniel et al. [43]. Daniel et al. [43] emphasize using a variety of methods (e.g., multiple-choice questions, objective structured clinical examinations, observation, global assessments and written notes) when assessing clinical reasoning competency which correspond to the learning environment (clinical and non-clinical); however, tests, multiple-choice questions and true–false questions are only rated from mediocre to weak in assessing information gathering, hypothesis generation and the representation of problems. Their strengths lie more in the assessment of differential diagnoses, principal diagnoses and management and treatment. Simulated clinical environment assessments are best for assessing information gathering, with direct observation and objective, structured clinical examinations being the strongest in this domain. Self-regulated learning strategies are effective tools for measuring hypothesis generation and problem posing because they force students to articulate these otherwise hidden steps in the reasoning process [43]. Furthermore, Daniel et al. [43] and Brown Tyo and McCurry [17] highlight the need for assessment methods to be valid and feasible. Brentnall et al. [44] also suggest that by carefully combining strategies that are strong in assessing the different components of clinical reasoning, educators can begin to ensure that all components of clinical reasoning are assessed. Despite this effort, this review also concluded that research is needed to develop, test and incorporate student assessments that are amenable to measuring outcomes in order to gain an understanding of student performance of this vital skill and how to support its development.

The category *clinical reasoning in real life* describes the understanding that clinical reasoning and decision making in nursing and midwifery is an interactional concept which involves not only nurses and midwives but also other members of interprofessional teams and emphasizes the need for sharing and exchanging information, opinions and conclusions about patients’ problems. The reason for such an understanding might reflect the involvement of nursing and midwifery in resolving collaborative patient problems [45] for which other team members are able to make decisions at a higher level (for example, physicians ordering diagnostics and treatment). Nurses and midwives constitute a distinct profession with their own identity and are empowered to make autonomous decisions that promote the care and well-being of patients. However, the participants discussed how the understanding of the autonomy of nursing and midwifery practice might be different in some of the participants’ countries due to contextual factors such as education, health service organization and work environment [46,47]. Inter- and intraprofessional work was also recognized as an important domain within clinical reasoning in previous studies [20,48,49]; it brings together different healthcare professionals to assess and diagnose patients more completely, reducing biases in reasoning and decision making and promoting patient-centered care in order to achieve better health outcomes. Gummesson et al. [44], using interprofessional accounts, made it possible to deepen students’ understanding of the essential skills for discussing, contrasting and calibrating their role in relation to other professional groups and the patient throughout a clinical reasoning process. This concept has the potential to highlight aspects that cannot be targeted in any other way. The scaffolding structure developed by Gummesson et al., which is based on clinical reasoning in a multi-professional context, seemed feasible for interprofessional interaction and collaboration in theoretical courses.

Another important finding of this study is that the holistic nature of nursing and midwifery was identified as an integral part of clinical reasoning and decision making. Clinical reasoning facilitates the definition of the nursing plan and allows its individualization, which has a significant impact on the effectiveness of the actions taken [14,50]. Participants emphasized that nurses and midwives reason and make decisions about patient problems in a much wider context than physicians. The reasons for this might be that nurses and midwives approach patient data holistically, spend more time with patients and monitor and further assess their condition, engage in problem solving at the bedside by carrying out countless orders regarding care, ensuring its quality, and, above all, anticipate the risk of emergencies. Furthermore, nurses’ and midwives’ practice is focused on providing patient-centered care [51,52].

### 4.1. Limitations and Trustworthiness

There are methodological considerations to take into account when determining the trustworthiness and transferability of findings. When considering credibility, we draw attention to the risk that the participants might have been influenced by each other’s responses; however, we believe that this would be only to a certain degree as we ensured that the facilitators allowed time for the participants to speak about or reflect on each discussion point. People were encouraged to write their thoughts, ideas and reflections down on paper; however, as we were using the World Café method as a technique for data collection, we were not able to assign data to individual participants. We also aimed to reduce the facilitators’ effect on the participants and the discussion itself by preparing the facilitators in advance and by developing a World Café question guide.

We also note that the convenience sampling could have affected the credibility of the findings; however, we believe that due to the heterogeneity of sample (different positions in nursing and midwifery education, research and practice), this would be only to a limited degree. Another challenge with convenience sampling was that our sample also included two participants with backgrounds in medicine and psychology. We recognize that they might have contributed to the World Café with different perspectives on clinical reasoning. However, we believe that involving two educators with extensive experience in nursing and midwifery education and research, and with different professional backgrounds than the majority of participants, would not significantly impact the credibility of the findings. Moreover, though we included participants from geographically and professionally diverse contexts, we recognize that the sample size is relatively small.

To increase neutrality in interpretation, we utilized a method of researcher triangulation, namely, we interpreted the data using two pairs of researchers independently and then resolved the differences. Furthermore, the interpretations were discussed with a member of the team through consultations. To ensure dependability and confirmability, we took steps to establish consistency in our documentation and interpretation of data (maintaining documentation of the analysis process—the code and theme development process and recording decisions and justifications).

We believe that the findings and interpretations from this study can be applicable and relevant to similar contexts.

### 4.2. Implications for Research and Education

This study highlights the need for a comprehensive analysis of the existing curricula for teaching clinical reasoning in the EU. Further understanding differences, opportunities and obstacles across various EU countries could provide an important insight for developing nursing and midwifery curricula which will respond to current and future global challenges. Moreover, exploring and developing methodologies for assessing clinical reasoning and implementing evidence-based assessment methods into nursing and midwifery curricula is crucial as it would allow for the education and training of competent and compassionate future nurses and midwives. Furthermore, it would also allow us to use limited resources rationally. This study also shed light on the potential benefits of interprofessional training in developing clinical reasoning skills. Future studies might further investigate whether integrating interprofessional learning improves clinical reasoning skills. The implications of this study could offer a valuable insight for educators and policy makers aiming to enhance clinical reasoning in nursing and midwifery across the EU.

## 5. Conclusions

In this study, we explored the understanding of clinical reasoning as a concept, experiences of teaching clinical reasoning, and practices related to using clinical reasoning in nursing and midwifery in five European Union countries. Our findings suggest that clinical reasoning is understood as a dynamic and complex cognitive process which is shaped by a range of knowledge, skills, experience and other meta competencies. We also noted that there is variation in the practices of teaching and learning clinical reasoning, with unclear evidence about the effectiveness of some of the teaching and assessment methods and techniques reported. Moreover, this study brought to light that clinical reasoning is practiced as a collaborative and interactional process involving the patient as a whole. The findings highlight the areas which can be improved in current nursing and midwifery education. This study’s findings can also inform the development of clinical reasoning in nursing and midwifery curricula and courses within and outside the project.

## Figures and Tables

**Table 1:** Overview of the theme, categories and subcategories.

Theme	Category	Subcategories
Learning a way of thinking	Spiral of thinking	Complexity Meta-skills and competency
	The learning and teaching of a way of thinking	Benefits and challenges Implementing clinical reasoning in curricula
	Clinical reasoning in real life	Levels and autonomy of decision making Clinical reasoning as an integral part of holistic care

## Data Availability

The data that support the findings of this study are available from the corresponding author upon reasonable request. Data are located in controlled-access data storage at the University of Ljubljana.

## Supplementary Materials

The following supporting information can be downloaded at: https://www.mdpi.com/article/10.3390/healthcare11222969/s1.

## Appendix A

Facilitator characteristics:

Experienced facilitators (one male, L.P., and three females, M.P., R.V. and M.R.) who are university lecturers and researchers with a nursing background led the discussion. Three of the facilitators hold a PhD degree. All were trained in qualitative research methods.

Question guide:

The participants answered semi-structured, open-ended questions. The World Café included opening questions such as (a) “What are your thoughts about what we are going to talk about?”, followed by (b) “What is clinical reasoning?” (examples of follow up questions: “How would you define clinical reasoning?” “What does it mean to you?” “Why Clinical is reasoning important?”); (c) “How do you teach CR? and “How do you assess learning outcomes?” (examples of follow up questions: “What helps or can be helpful at teaching CR?” “What helps or can be helpful in assessing learning outcomes?” “What are (can be) challenges in teaching CR?” “What are (can be) challenges in assessing learning outcomes?”); and (d) “In which areas of clinical practice and how do you believe nurses/midwives could effectively utilize clinical reasoning?” (examples of follow up questions: “Please give some examples of where and how could CR be used?” “What specific challenges or situations within those areas do you think would benefit the most from the using of clinical reasoning?”).
